# A multiparametric perspective on C6 and F98 cell lines in orthotopic rat models for glioblastoma research

**DOI:** 10.1038/s41598-025-06684-5

**Published:** 2025-07-02

**Authors:** Carlos Caro, Nuria Arias-Ramos, Jesús David Urbano-Gámez, Raquel González-Alday, Pilar López-Larrubia, María Luisa García-Martín

**Affiliations:** 1 Biomedical Magnetic Resonance Laboratory-BMRL, Andalusian Public Foundation Progress and Health-FPS, Seville, Spain; 2 Instituto de Investigación Biomédica de Málaga y Plataforma en Nanomedicina - IBIMA Plataforma BIONAND, C/ Severo Ochoa, 35, 29590 Málaga, Spain; 3 Instituto de Investigaciones Biomédicas Sols-Morreale (CSIC-UAM), Arturo Duperier, 4, 28029 Madrid, Spain; 4 Biomedical Research Networking Centre on Rare Diseases (CIBERER), Institute of Health Carlos III, Madrid, Spain; 5 Instituto de Investigación Sanitaria La Paz (IdiPaz), Madrid, Spain; 6 Biomedical Research Networking Center in Bioengineering, Biomaterials and Nanomedicine (CIBER-BBN), 28029 Madrid, Spain

**Keywords:** MRI, MRS, Multiparametric imaging, Glioblastoma, Orthotopic tumor models, Host-tumor interaction, Cancer, Cell biology, Neuroscience, Biomarkers

## Abstract

Glioblastoma remains a significant healthcare challenge due to its aggressiveness and poor prognosis, underscoring the urgent need for innovative approaches to tackle this disease. While there is a growing emphasis on the use of in vitro models for these developments, animal models continue to play a pivotal role, as they provide insights into the complex tumor microenvironment (TME) and host interactions that in vitro systems cannot fully replicate. However, selecting the appropriate animal model based on the research question is fundamental to ensure the reliability and translational potential of preclinical findings. Advanced neuroimaging techniques, particularly magnetic resonance imaging (MRI) and spectroscopy (MRS), offer unique advantages for the in vivo characterization of glioblastoma models. These techniques offer comprehensive morphological, functional, and metabolomic insights, enabling the evaluation of critical tumor features, such as vascular permeability, infiltrative capacity, or metabolism, among others. In this study, different glioblastoma models were generated by orthotopic implantation of two widely used glioblastoma cell lines, C6 and F98, in Fischer, Sprague Dawley, and Wistar rats and characterized by multiparametric MRI, MRS, along with histological analysis. Results revealed that the F98-Fischer model closely mimics human glioblastoma regarding vascular permeability, infiltrative growth, and key metabolic hallmarks. In contrast, the C6-Wistar model, while exhibiting similarities in permeability and metabolism, lacked invasive growth. Moreover, the two cell lines showed different responses to the host environment, with F98 consistently forming infiltrative tumors across rat strains, while C6 showed variability in tumor growth patterns, suggesting a greater dependence on the host microenvironment. These findings underscore the complex interplay between cell line genetics and host factors in defining tumor phenotype, emphasizing the importance of considering host-tumor interactions in glioblastoma research.

**Supplementary Information:**

The online version contains supplementary material available at 10.1038/s41598-025-06684-5.

## Introduction

According to the World Health Organization (WHO), as of 2022, cancer remained one of the most significant global health crises, consistently ranking among the top five leading causes of death worldwide^1^. Among primary tumors of the central nervous system (CNS), gliomas are the most frequently diagnosed, with malignancy levels ranging from low-grade (grade I) to highly aggressive (grade IV)^2,3^. Glioblastoma, a grade IV glioma, is the most common and aggressive malignant brain tumor, representing 15% of all malignant CNS tumor cases globally^4^. It poses a significant challenge due to its highly aggressive nature and grim prognosis, with a median survival of approximately 15 months and an overall 5-year survival rate of only ≈ 5%^5^. Glioblastoma diagnosis relies on the so-called “integrated diagnosis,” which combines neuroimaging exams with histological and molecular information^6^. Magnetic resonance imaging (MRI) is the method of choice for the preoperative assessment of CNS tumors. It stands out for its particular combination of qualities, highlighting its non-invasive character, the absence of ionizing radiation, and excellent image quality, providing relevant information about tumor structure/composition, physiology, hemodynamics, and microenvironment at the voxel level^7^, along with metabolic profiling^8^. In this context, advanced neuroimaging exams, including dynamic contrast enhancement (DCE), perfusion-weighted imaging (PWI), diffusion-weighted imaging (DWI), diffusion tensor imaging (DTI), and spectroscopy (MRS), have considerably improved glioblastoma diagnosis accuracy^9–11^.

Cancer models aim to address key questions, primarily understanding molecular pathways in tumorigenesis and assessing the effectiveness of current or novel therapeutic strategies. Although in vitro models have long been advocated as a plausible alternative to animal models, they are limited in their ability to mimic tumor heterogeneity and its microenvironment, which hinders a comprehensive understanding of tumor pathogenesis, therapeutic responses, and adverse reactions^12^. Therefore, animal tumor models, particularly rodent models, remain an invaluable tool in cancer research, either in exploring their genetic/molecular basis or in testing new diagnostic/therapeutic compounds^13^. Most commonly used glioblastoma animal models are based on the orthotopic implantation of established cell lines, either rodent or human. The former are typically implanted in immunocompetent animals, whereas the latter are implanted in immunodeficient animals. However, immunodeficient animals have limitations, hindering the study of immunotherapies and the evaluation of the role of immunity/inflammation in cancer growth^14^. Although the molecular mechanisms linking immunity and inflammation to cancer remain elusive, growing evidence suggests that the inflammatory microenvironment plays a pivotal role in tumor development^15^. Therefore, animal cell lines, which can be developed in immunocompetent recipients, continue to play an important role in cancer research. Some of the most commonly used animal glioblastoma models in immunocompetent animals are C6 and F98 cell lines implanted in their original animal strain, Wistar and Fischer rats, respectively^16–18^. However, although both models have been widely used as immunocompetent glioblastoma models, their immunogenic response is significantly different^19^. The C6 model was developed in outbred Wistar rats and, as a result, behaves as an allogenic model, eliciting a high immunogenic response even with a low number of implanted cells^20^. In contrast, the F98 model, derived from inbred Fischer 344 rats, is a syngeneic model when implanted into the same rat strain, leading to low immunogenicity^21^. From a genetic perspective, both cell lines share several genetic features typically found in human glioblastomas, such as p53 mutation or epithermal growth factor receptor (EGFR) overexpression, among others. However, they also show genetic differences, such as overexpression of insulin-like growth factor-I (IGF-I), which was only found in C6, or the overexpression of cyclins D1 and D2, only present in F98 cells^19,22,23^.

Interestingly, tumors derived from the orthotopic implantation of these cell lines showed significant differences in their metabolic profile and growth pattern. Thus, in vivo MRS profiles showed decreased levels of total creatine (tCr), which corresponds to the sum of overlapping creatine (Cr) and phosphocreatine (PCr), and increased levels of lactate in F98 tumors with respect to the contralateral brain parenchyma, whereas no variations in these two metabolites were observed in C6 tumors. In contrast, C6 tumors showed decreased levels of taurine (Tau), while F98 tumors showed no variation. Moreover, both models showed, as expected, decreased levels of *N*-acetyl aspartate (NAA), while total choline (tCho), which corresponds to the sum of overlapping choline, phosphocholine (PCho), and glycerophosphocholine (GPC), remained unchanged^17^.

As for their growth pattern, C6 tumors are moderately aggressive, with a distinct peritumoral region, and preferentially use existing blood vessels to support their growth. In contrast, F98 tumors are more invasive, exhibiting a highly infiltrative growth pattern and more pronounced vascular alterations^24^. Additionally, both tumor models typically show necrosis, whereas peritumoral edema is more frequently observed in F98 tumors^17^.

Whether these differences in morphology and metabolism result from their distinct genetic backgrounds or from interactions with the host microenvironment, potentially involving immunogenicity, remains unclear.

Remarkably, C6 tumors grown in various mouse strains have been shown to recapitulate the glioblastoma features of the original model^25^. Indeed, C6 tumor-bearing immunocompetent mice have been utilized as a glioblastoma model in several studies^26,27^. However, to our knowledge, similar studies have not been conducted with F98.

In this work, we aimed to shed light on the effect of the host on tumor growth patterns and metabolism by implanting C6 and F98 cells within the three most commonly used rat strains, Wistar, Fischer, and Sprague Dawley (SD), and performing a comprehensive characterization study using in vivo multiparametric MRI and MRS, and hystological analysis. Specifically, we determined a wide range of variables, including T_2_-WI, K_trans_, V_e_, DTI parameters, such as mean diffusivity (MD) and fractional anisotropy (FA), magnetic transfer ratio (MTR), and local metabolism, and compared them to data from human glioblastomas extracted from the literature. These MRI parameters provide valuable information about different features of the TME^28^. T_2_ values are related to water content within a tissue, while MD provides information on the motion restriction of the water molecules, thereby being essential for evaluating the presence and type of edema. FA allows for the evaluation of the integrity and organization of white matter fibers, which, in the case of brain tumors, is particularly useful for assessing tumor invasiveness. MTR values are associated with macromolecular content, which allows for effective differentiation of necrotic areas from viable parts of the tumor and edema. Finally, MRI findings were validated by histological analysis, including myelin staining with Luxol fast blue (LFB).

## Materials and methods

### Animals

Male rats of different strains (Wistar, Fischer, and Sprague Dawley) of 8 weeks (190 g, n = 8 per group, including technical outliers) were used for in vivo experiments. These experiments were conducted in accordance with the Spanish and European Guidelines for Care and Use of Laboratory Animals (R.D. 53/2013 and 2010/62/UE) and approved by our local ethical committee and the Highest Institutional Ethical Committee (Andalusian Government, accreditation number 14/09/2021/129). Additionally, we endeavored to conduct this study with adherence to the ARRIVE guidelines (Animal Research: Reporting of In Vivo Experiments) as outlined by the National Centre for the Replacement, Refinement and Reduction of Animals in Research (NC3Rs).

### Tumor implantation

#### Cell culture

Two different rat glioblastoma cells were selected as working models: C6 and F98. Both cell lines were purchased from the American Type Culture Collection (ATCC). Additional details regarding cell culture conditions are provided in the supplementary information (SI).

#### Stereotactic implantation

Both C6 and F98 cells (passages 5–10) were brought to 80–90% confluence in culture. Then, cells were trypsinized, pelleted, and stored at 4 °C while rats were anesthetized and prepared for implantation (approximately 20 min). Rats were anesthetized intraperitoneally with a mixture of anesthetics (0.1 mg/kg Buprenorphine/0.5 mg/kg Medetomidine/50 mg/kg Ketamine) in 500 µl of sterile water. Subsequently, they were placed in the stereotactic apparatus, exposing the skull by an incision in the skin. A small perforation was performed 3.5 mm to the right of Bregma. 10^5^ cells of either C6 or F98 line were inoculated at a depth of 5.5 mm from the cranial surface, specifically targeting the caudate nucleus, as previously described^29^. Post-surgery, the drinking water was supplemented with buprenorphine at a concentration of 0.056 mg/ml to serve as an analgesic method.

### Magnetic resonance studies

Both MRI and MRS experiments were carried out on a Bruker Biospec 9.4 T system (Bruker BioSpin GmbH, Ettlingen, Germany) equipped with 400 mT/m gradients, using a 72 mm volume resonator for excitation and a 20 mm inner-diameter surface coil for signal reception placed on top of the head of the rat. Experiments were acquired using ParaVision 6.1. software (Bruker BioSpin GmbH, Ettlingen, Germany) operating on a Linux platform. A comprehensive description of the experimental scheme, sequences, and image processing/analysis is available in the SI.

### Histology

After the MR experiment, animals were sacrificed, and various tissues were harvested for histological evaluation. Haematoxylin and Eosin (H&E) and Luxol Fast Blue (LFB) stainings were used to assess tissue architecture and observe myelin in the central nervous system (CNS), respectively. Protocols are described in detail in the SI.

### Statistical analysis

Statistical analysis was conducted using SPSS version 26. For comparisons between animals of the same strain implanted with different cell types, the Mann–Whitney U-test was applied. To assess differences among animals of different strains implanted with the same cell type, the Kruskal–Wallis test, followed by Dunn’s test, was employed. *P* values lower than 0.05 were considered to be statistically significant.

Graphical representations were generated using GraphPad Prism Software, version 9 (GraphPad Software, La Jolla, CA, USA). Data obtained from the parametric maps were represented as boxplots, where the horizontal bar represents the median, the '‘+’ symbol shows the mean, and the lower and upper limits of the box indicate the first and third quartiles, respectively. The upper and lower whiskers extend to the most extreme data points 1.5 × the interquartile range from the nearest box border (quartile). Outliers, if present, are indicated by the ‘·’ symbol. Statistically significant values (*p* < 0.05) were indicated by ‘*’ symbol. Outliers were defined using the interquartile range (IQR) method. Specifically, values were considered outliers if they exceeded three times the IQR above the third quartile (Q3 + 3 × IQR) or below the first quartile (Q1 − 3 × IQR). Values between 1.5 × IQR and 3 × IQR from the quartiles were retained.

Spearman correlation coefficients (SCC) were calculated between samples considering all metabolite concentrations. The data were represented in a correlation matrix using the free software JAMOVI (v 2.3.21).

The SCC values are provided as a measure of similarity between samples within each group and interpreted as a comparative measure of homogeneity. For this analysis, each individual is treated as a variable, and their metabolic profile (absolute metabolite concentrations) is used as a set of values for that variable. Thus, the resulting SCC reflects the degree of similarity between metabolic profiles across individuals within the same group. This is possible because the order of metabolites is kept constant among individuals. To the best of our knowledge, while correlation matrices are commonly used in metabolomics to explore associations and system-level relationships^30^, the specific application of the mean SCC as a quantitative measure of sample homogeneity has not been widely reported in metabolomics. However, this approach has been previously used to analyze similarity between replicates in proteomics^31,32^.

## Results

Six different orthotopic rat tumor models (ORMs), C6-Wistar, C6-Fischer, C6-SD, F98-Wistar, F98-Fischer, and F98-SD, were developed and characterized by multiparametric magnetic resonance exams and histological analysis.

### Anatomical MRI and dynamic contrast enhancement (DCE)

**Fig. 1:**
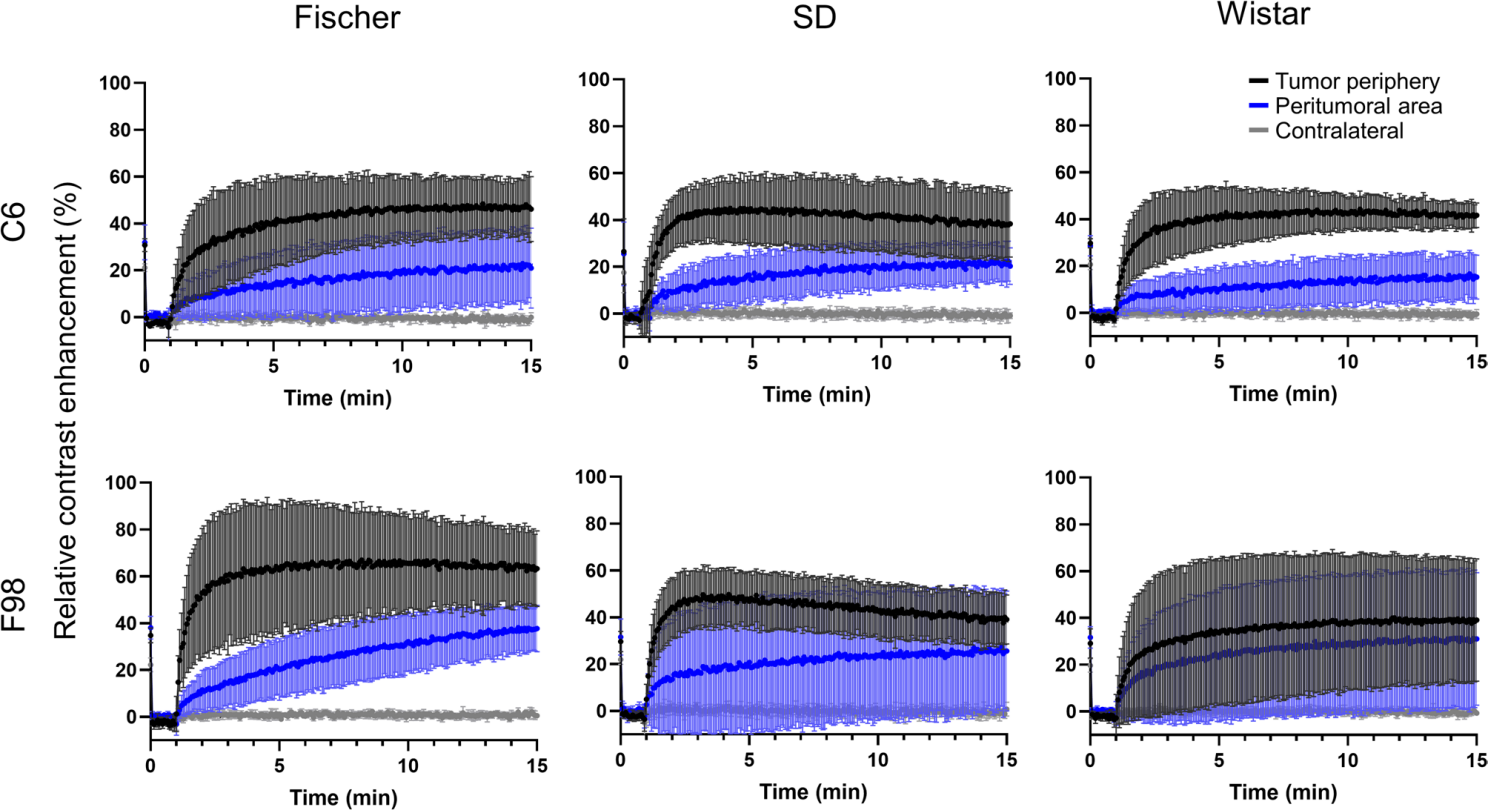
Relative contrast enhancement (RCE) curves (mean ± standard deviation) of the tumor periphery, peritumoral area, and contralateral region of experimental groups calculated from DCE studies of the different ORMs studied.

Tumor lesions typically exhibit heterogeneity on T_2_-weighted images (T_2_WI), whether comparing different tumor zones or animals (Fig. S1). For quantitative analysis of the DCE-MRI curves, three different zones were defined based on T_1_-weighted images (T_1_WI) acquired 1.5 min after gadolinium-based contrast agent (CA) administration (Fig. S1): the tumor periphery, where the CA can easily extravasate (hyperintense regions); the peritumoral area, where CA extravasation could be taking place due to tumor invasion of the healthy tissue (slightly hyperintense regions); and a region of equivalent size on the contralateral hemisphere. Tumor necrosis-appearing regions (hypointense regions within the tumor) were excluded from this analysis due to their intrinsic heterogeneity. Of note, no hypointense tumor regions were present in the C6-Fischer group. DCE curves analysis showed fast CA uptake in the tumor periphery for C6-Wistar, C6-SD, F98-Fischer, and F98-SD (Fig. 1), reaching the maximum relative contrast enhancement (time-to-peak) within 4–7 min (Table 1). Moreover, all these groups presented a positive wash-in slope, measured between 1 and 1.8 min post-acquisition start, from 30 to 50 relative contrast enhancement/min (% RCE/min), and a moderate negative wash-out slope during the 3 last minutes (Table 1). On the contrary, C6-Fischer and F98-Wistar showed a slow CA uptake, with a time-to-peak of around 10 min or even longer, and a steady state up to the end of the experiment (Fig. 1 and Table 1). Notably, the DCE-MRI curves of the F98-Wistar group exhibited extremely high heterogeneity. On the other hand, there were no significant differences in the peritumoral area among the models, not reaching the maximal enhancement during the experimental time, and with a high degree of heterogeneity in the DCE curves. Finally, no CA uptake was observed in the contralateral hemisphere in any case.

**Table 1:** Summary of the different parameters determined from the MRI experiments.

	F98-fischer	F98-SD	F98-wistar	c6-fischer	C6-SD	C6-wistar
Tumor periphery						
Time-to-peak (min)	6.7 ± 4.7	3.6 ± 1.0	11.1 ± 5.6	9.7 ± 5.1	3.7 ± 1.4	7.0 ± 4.5
Wash-in slope	52.5 ± 25.0	40.1 ± 10.9	29.1 ± 28.1	30.2 ± 19.2	32.3 ± 13.9	32.4 ± 13.9
Wash-out slope	-0.6 ± 1.0	− 0.4 ± 0.5	0.4 ± 0.9	0.4 ± 1.0	− 0.9 ± 0.3	− 0.4 ± 1.,0
K_trans_ (min^−1^)	0.20 ± 0.04	0.24 ± 0.11 (*)	0.12 ± 0.11 (*)	0.19 ± 0.14	0.12 ± 0.03	0.14 ± 0.08
V_e_	0.52 ± 0.13	0.28 ± 0.10	0.36 ± 0.18	0.61 ± 0.29	0.35 ± 0.14	0.43 ± 0.09
T_2_ (ms)	55.3 ± 3.7	50.3 ± 3.8	58.9 ± 6.6 (*)	56.6 ± 5.8	53.7 ± 3.7	52.3 ± 4.6
MD (μm^2^ s^−1^)	1261.2 ± 211.1	1031.0 ± 150.6	1531.2 ± 387.5 (*)	1250.3 ± 249.6	1114.8 ± 177.7	1217.5 ± 229.3
FA	0.18 ± 0.03	0.19 ± 0.04	0.25 ± 0.10	0.17 ± 0.02	0.24 ± 0.12	0.23 ± 0.07
MTR (%)	25.3 ± 2.5	27.3 ± 0.9	25.5 ± 2.7	24.7 ± 3.3	25.0 ± 2.4	25.0 ± 2.6
Peritumoral area						
Time-to-peak	13.1 ± 0.4	12.1 ± 2.0	12.1 ± 1.8	12.8 ± 1.1	11.2 ± 3.1	13.0 ± 0.8
Wash-in slope	10.5 ± 9.3	10.7 ± 17.3	13.9 ± 20.0	6.9 ± 9.0	7.4 ± 9.3	6.4 ± 4.7
Wash-out slope	0.7 ± 0.4	0.3 ± 0.9	0.2 ± 0.5	0.8 ± 0.4	0.2 ± 0.7	0.6 ± 0.4
K_trans_	0.04 ± 0.03	0.05 ± 0.08	0.07 ± 0.08	0.05 ± 0.04	0.03 ± 0.03	0.05 ± 0.08
V_e_	0.39 ± 0.23	0.17 ± 0.17	0.26 ± 0.23	0.33 ± 0.21	0.21 ± 0.11	0.24 ± 0.17
T_2_ (ms)	68.2 ± 9.8 (*)	63.4 ± 14.7	63.7 ± 13.0 (*)	53.5 ± 8.7	56.7 ± 6.8	45.6 ± 3.6 (*)
MD (μm^2^ s^−1^)	1807.3 ± 322.1 (*)	1548.7 ± 405.1	1663.1 ± 410.6 (*)	1297.3 ± 305.3	1335.7 ± 303.4	1167.4 ± 231.5
FA	0.19 ± 0.04	0.20 ± 0.08	0.25 ± 0.11	0.24 ± 0.05	0.26 ± 0.09	0.30 ± 0.06
MTR (%)	24.9 ± 2.7	28.0 ± 7.6	26.5 ± 4.1 (*)	29.0 ± 5.0	23.1 ± 4.7	31.6 ± 3.5 (*)

(*) statistically significant (*p* < 0.05).

**Fig. 2:**
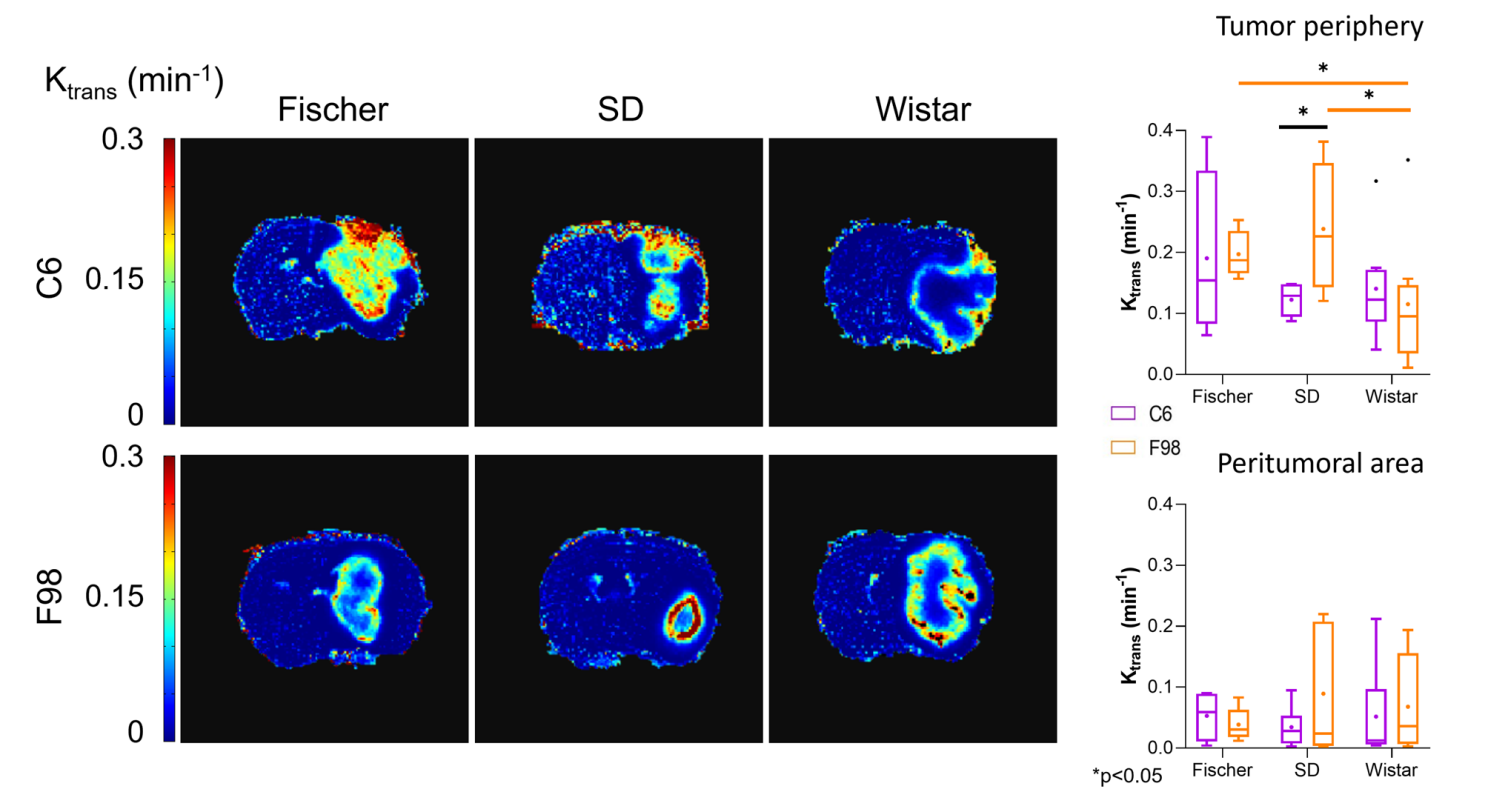
Analysis of K_trans_ maps obtained from DCE images. On the left, K_trans_ maps from a representative rat of each ORM studied. On the right, quantification of K_trans_ data from tumor periphery and peritumoral/edema area of the different ORMs represented as boxplots (*p < 0.05, n ≥ 5).

Additionally, other parameters were calculated from DCE-MRI experiments (Fig. S2), namely, the volume transfer constant (K_trans_) and the extravascular-extracellular space volume fraction (V_e_). The K_trans_ value is known to be strongly associated with the pathological grade of gliomas^33^. The K_trans_ from the tumor periphery areas was statistically different (*p* < 0.05) between F98-SD and C6-SD, with the latter showing the lowest value. Statistically significant differences were also found between F98-Wistar and either F98-SD or F98-Fischer (Fig. 2), with F98-Fischer having the lowest value. Lower K_trans_ values were observed in the peritumoral region, indicating less contrast extravasation; however, no statistical differences were found in this region among the experimental groups.

Regarding V_e_, no significant differences were observed for any of the analyzed zones (Fig. S3).

### T_2_-mapping

**Fig. 3:**
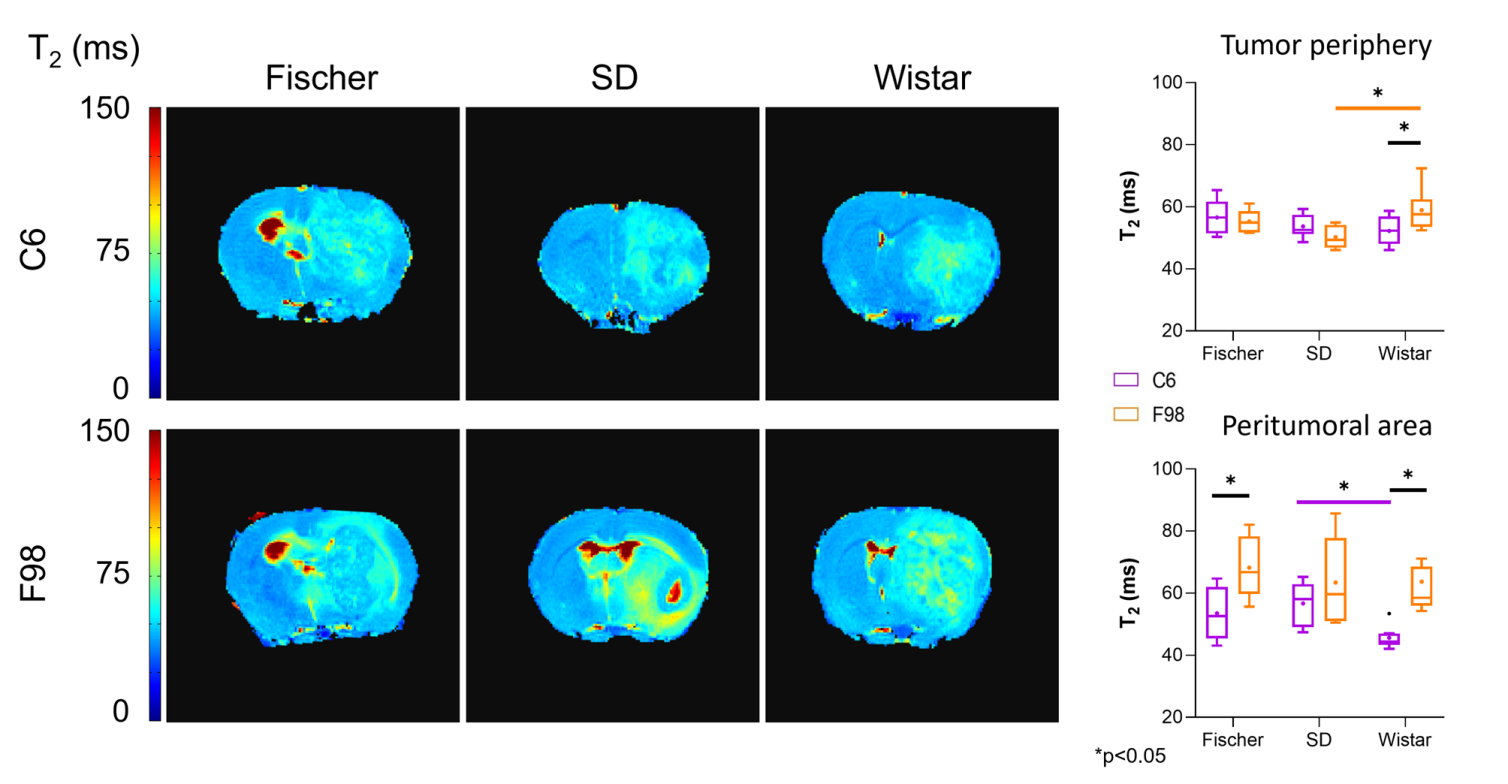
Analysis of T_2_ maps. On the left, T_2_ maps from a representative rat of each ORM studied. On the right, quantification of T_2_ data from tumor periphery and peritumoral/edema area of the different ORMs represented as boxplots (* *p* < 0.05, n ≥ 5).

This parameter was evaluated exclusively on the tumor periphery and peritumoral region, as both areas are relevant to tumor growth and potential expansion (Fig. 3). Notably, the F98-Wistar group exhibited significantly higher average T_2_ values in the tumor periphery (*p* < 0.05) compared to the C6-Wistar and F98-SD groups. Regarding the peritumoral area, higher T_2_ values were observed in the animals implanted with F98 cells than in animals implanted with C6 cells across all rat strains, being statistically significant (*p* < 0.05) in Fischer and Wistar strains, not in SD. Similar values were observed in the three groups implanted with F98 cells and in those with C6 cells, with significant differences found only between C6-SD and C6-Wistar.

### Diffusion tensor imaging (DTI)

**Fig. 4:**
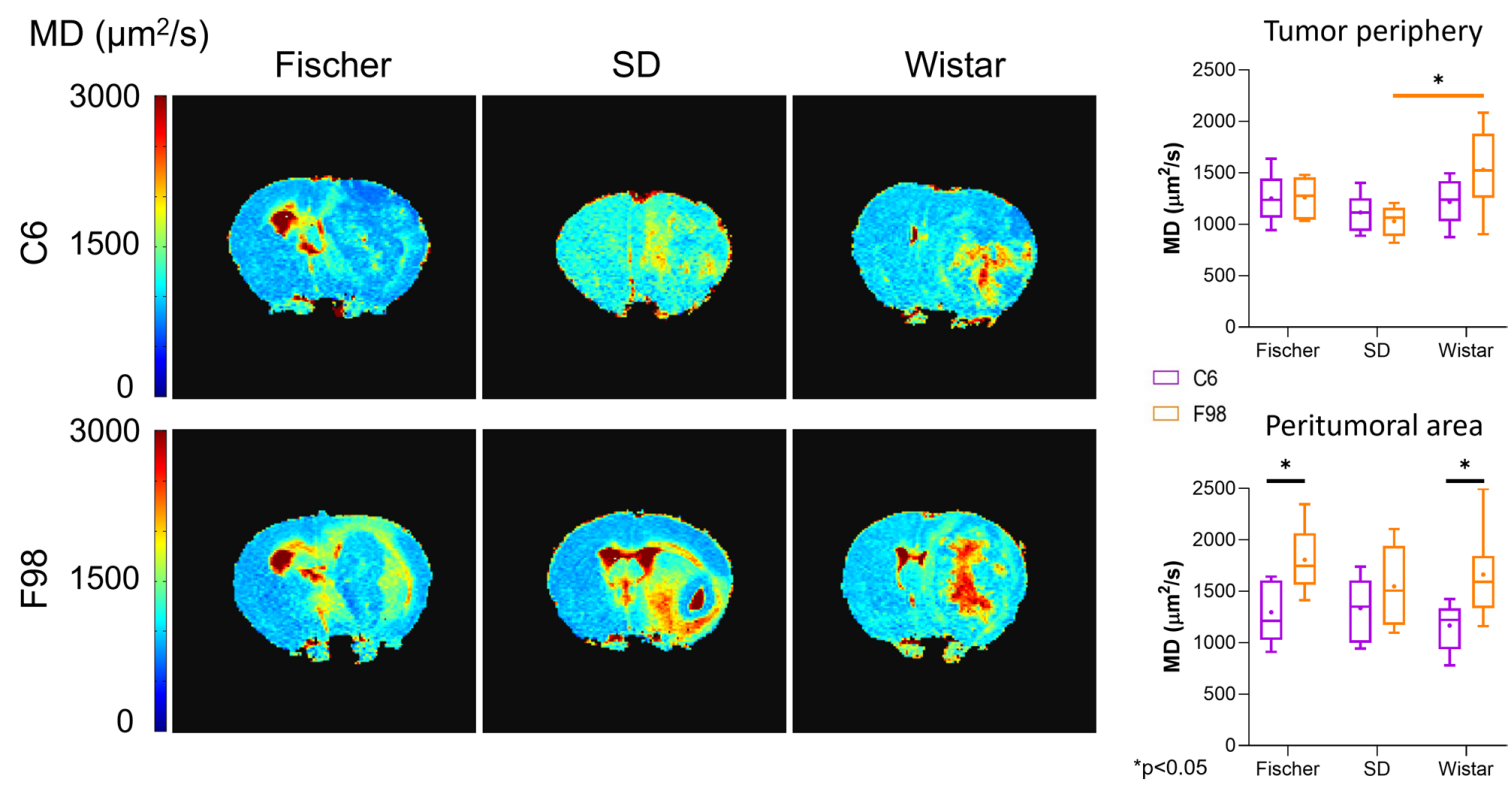
Analysis of **A23**. maps from DTI studies. On the left, MD maps from a representative rat of each ORM studied. On the right, quantification of MD data from the tumor periphery and peritumoral/edema areas of the different ORMs represented as boxplots (*p < 0.05, n ≥ 5).

Two parameters were obtained and analyzed from the DTI experiments: the mean diffusivity (MD), which reflects the overall degree of water diffusional freedom within a tissue, and the fractional anisotropy (FA), a scalar parameter reflecting the degree of anisotropy in water diffusion. In the analysis of the tumor periphery region (Fig. 4), similar to the T_2_ analysis, the F98-Wistar group exhibited greater differences with increased MD. This increase showed a trend compared to C6-Wistar (*p* = 0.1) and was statistically significant compared to F98-SD (*p* < 0.05). Regarding the peritumoral area, similar results to T_2_ were observed, with statistically significant higher values (*p* < 0.05) in rats implanted with F98 in both Fischer and Wistar rats. Moreover, no statistically significant variations in FA were observed in the tumor periphery region or the peritumoral area (Fig. S4); yet, a trend was noted in the peritumoral area of Fischer rats, with higher values in C6-Fischer animals compared to F98-Fischer rats (*p* = 0.07).

### Magnetic transfer ratio (MTR)

Similar MTR values were observed among groups in the tumor periphery (Fig. S5). Conversely, in the peritumoral area, statistically significantly higher MTR values were found in C6-Wistar rats compared to F98-Wistar rats. Moreover, among rats implanted with F98 cells, statistically significant higher MTR values were observed in SD rats compared to Wistar rats.

The parameters derived from the MRI studies indicated above, namely time-to-peak, wash-in slope, wash-out slope, K_trans_, V_e_, T_2_, MD, FA, and MTR, obtained from the different ORMs and the two analyzed zones, are summarized in Table 1.

### Magnetic resonance spectroscopy (MRS)

MRS is able to accurately detect and quantify several metabolites in vivo, enabling noninvasive evaluation of both tumor and healthy brain metabolic profiles (Fig. S6). Detectable metabolites include NAA, tCr (creatine + phosphocreatine), *myo-*inositol + glycine (mI + Gly), glutamate (Glu), glutamine (Gln), lactate, tCho (choline + phosphocholine + glycerophosphocholine), glutathione, and Tau. Significant differences (*p* < 0.05) or trends (p < 0.1) were observed between the glioblastoma metabolic profile and the contralateral hemisphere for both C6 and F98 tumors in the different strains (Table 2). Unexpectedly, NAA only displayed a statistically significant decrease (*p* < 0.05) in the tumor tissue of C6-Fischer and C6-SD. Likewise, tCr showed the same variation, with a statistically significant decrease (*p* < 0.05) in C6-Fischer, C6-SD, and also F98-Wistar. mI + Gly was significantly increased in the tumor tissue of F98-Fischer and C6-Wistar. Furthermore, as expected, all groups showed a significant increase (*p* < 0.05) in the tCho levels in the tumor tissue compared to the contralateral tissue. However, no significant changes were detected in any case for Glu and glutathione. For Gln, an increasing trend was detected in the tumor tissue for F98-SD, along with a statistically significant increase in C6-Fischer and C6-Wistar. Lactate exhibited a significant increase in F98-Fischer, C6-SD, and F98-SD tumor tissue. Finally, Tau showed an increasing trend (*p* < 0.1) in the tumor tissue of F98-SD and a significant increase (*p* < 0.05) in F98-Fischer and F98-Wistar.

**Table 2:** Metabolite concentrations measured in vivo by localized ^1^H MRS. Concentrations are provided in mM, with values corresponding to the median and interquartile range (in parentheses).

Cell line	F98									C6								
Rat Strain	Fischer			SD			Wistar			Fischer			SD			Wistar		
Region	Tumor	Contralat	T vs C	Tumor	Contralat	T vs C	Tumor	Contralat	T vs C	Tumor	Contralat	T vs C	Tumor	Contralat	T vs C	Tumor	Contralat	T vs C
Glu	14.5 (3.2)	11.1 (2.7)	ns	14.4 (6.6)	11.7 (1.9)	ns	9.4 (4.5)	10.6 (4.0)	ns	11.6 (1.7)	13.1 (4.7)	ns	13.6 (6.3)	17.6 (2.3)	ns	12.2 (3.4)	13.1 (5.5)	ns
Glu + Gln	18.0 (2.2)	13.9 (2.6)	↑ #	20.2 (5.9)	14.5 (1.8)	↑ #	13.5 (6.8)	14.2 (3.7)	ns	14.7 (2.0)	15.3 (4.4)	ns	16.7 (4.9)	19.8 (1.9)	↓ #	17.3 (4.0)	15.8 (5.1)	ns
Glutathione	2.6 (1.1)	2.1 (0.6)	ns	2.8 (0.4)	2.1 (0.2)	ns	2.4 (1.2)	2.6 (0.8)	ns	2.1 (0.4)	2.24 (0.3)	ns	3.2 (1.4)	2.6 (1.6)	ns	2.6 (1.1)	2.5 (0.1)	ns
Lactate	11.4 (4.3)	0.0 (0.0)	↑ ***	19.0 (16.6)	0.0 (0.0)	↑ **	0.0 (8.3)	0.0 (0.8)	ns	5.0 (16.0)	0.0 (0.0)	ns	15.1 (7.5)	0.0 (3.6)	↑ *	2.8 (9.0)	0.00 (2.7)	ns
Tau	19.6 (7.3)	9.6 (2.9)	↑ ***	17.2 (14.9)	8.7 (2.4)	↑ #	18.8 (7.8)	12.8 (3.5)	↑ *	14.6 (3.9)	12.7 (0.4)	ns	16.5 (12.8)	15.2 (2.7)	ns	21.4 (10.1)	15.9 (2.0)	ns
tCho	3.1 (0.7)	1.3 (0.2)	↑ ***	3.4 (1.0)	1.1 (0.2)	↑ *	2.5 (1.0)	1.4 (0.4)	↑ **	3.1 (1.1)	1.4 (0.1)	↑ *	4.4 (1.6)	1.8 (0.3)	↑ **	2.9 (0.6)	1.4 (0.4)	↑ ***
NAA	5.4 (1.9)	6.8 (2.4)	ns	4.0 (4.9)	6.1 (0.5)	ns	4.7 (3.8)	7.8 (2.9)	ns	2.5 (1.2)	7.4 (1.0)	↓ *	5.4 (3.0)	9.7 (0.9)	↓ **	7.4 (3.2)	9.7 (2.3)	ns
mI + Gly	4.8 (1.4)	2.9 (1.1)	↑ **	5.8 (2.6)	3.6 (0.6)	ns	5.2 (1.8)	4.6 (1.5)	ns	5.4 (2.7)	4.1 (0.5)	ns	7.0 (3.7)	5.6 (1.1)	ns	6.1 (1.1)	4.7 (0.6)	↑ *
tCr	6.1 (2.4)	7.0 (1.3)	ns	4.7 (1.0)	6.1 (1.3)	ns	5.3 (2.5)	7.8 (2.0)	↓ **	4.5 (1.2)	7.6 (0.4)	↓ *	7.7 (2.4)	9.3 (1.4)	↓ *	7.5 (2.0)	8.7 (2.0)	ns
Gln	2.8 (0.6)	2.0 (0.6)	ns	3.8 (2.7)	2.2 (1.2)	↑ #	3.2 (3.0)	2.3 (1.1)	ns	2.5 (0.5)	2.1 (0.1)	↑ *	2.6 (1.1)	2.8 (0.6)	ns	3.9 (1.2)	2.4 (0.6)	↑ **

Statistical significance: #*p* values < 0.1; *p values < 0.05; **p values < 0.01; ***p values < 0.005. *ns* not statistically significant.

**Fig. 5:**
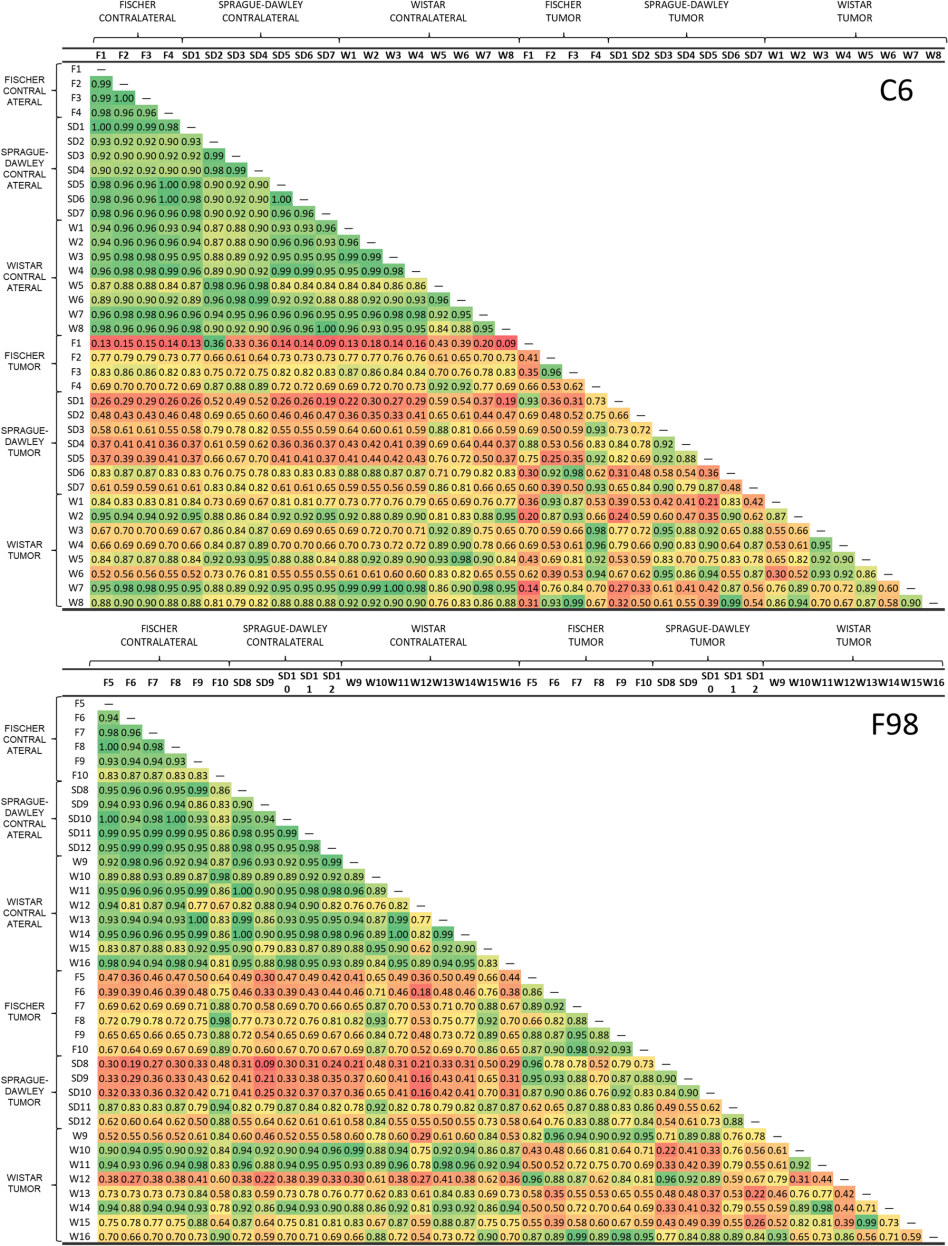
Correlation matrix of metabolomics profiles. Correlation matrix computed using the SCC between individuals for C6 (left) and F98-bearing (right) models (n ≥ 5). The color scale represents values between 1 (green) and 0 (red). Contralateral tissues are grouped in the upper and left sides of the matrix, and tumors are in the bottom and right parts of the matrix.

A correlation matrix based on the Spearman correlation coefficient was generated among individuals (Fig. 5) to compare metabolic profiles. The SCC defines the correlation between two variables considering the value for this variable in each individual, the monotonic relationship of the values, their rank difference, and relative order. Conventionally, this approach has been used to define the correlation between different variables, in this case, metabolites whose values correspond to the different concentrations across samples within the experiment. The SCC reflects the similarity of metabolic profiles based on concentrations. Typically, this value is high because the relative differences in metabolite concentrations tend to be similar across individuals. However, metabolic changes can alter these rankings, leading to lower SCC values and indicating reduced similarity in metabolic profiles. As observed, there is high homogeneity among contralateral tissues (for F98, SCC mean: 0.918 ± 0.064; for C6, SCC mean: 0.939 ± 0.041), with distinct differences in tumors (for F98 vs. contralateral, SCC mean: 0.646 ± 0.212; for C6 vs. contralateral, SCC mean: 0.677 ± 0.215). Notably, the most significant differences between the tumor and the contralateral tissue were observed in SD models (for F98, SCC mean: 0.472 ± 0.226; for C6, SCC mean: 0.579 ± 0.183). Conversely, these differences were lower in the F98-Wistar (SCC mean: 0.746 ± 0.188) and C6-Wistar models (SCC mean: 0.816 ± 0.117). Furthermore, some heterogeneity was observed among tumors, with the F98-Fischer (SCC mean: 0.879 ± 0.072) and C6-Wistar (SCC mean: 0.751 ± 0.167) models demonstrating the highest homogeneity. It is important to note that this correlation compares profiles; therefore, differences in metabolite concentrations among individuals may not be reflected by the SCC.

**Fig. 6:**
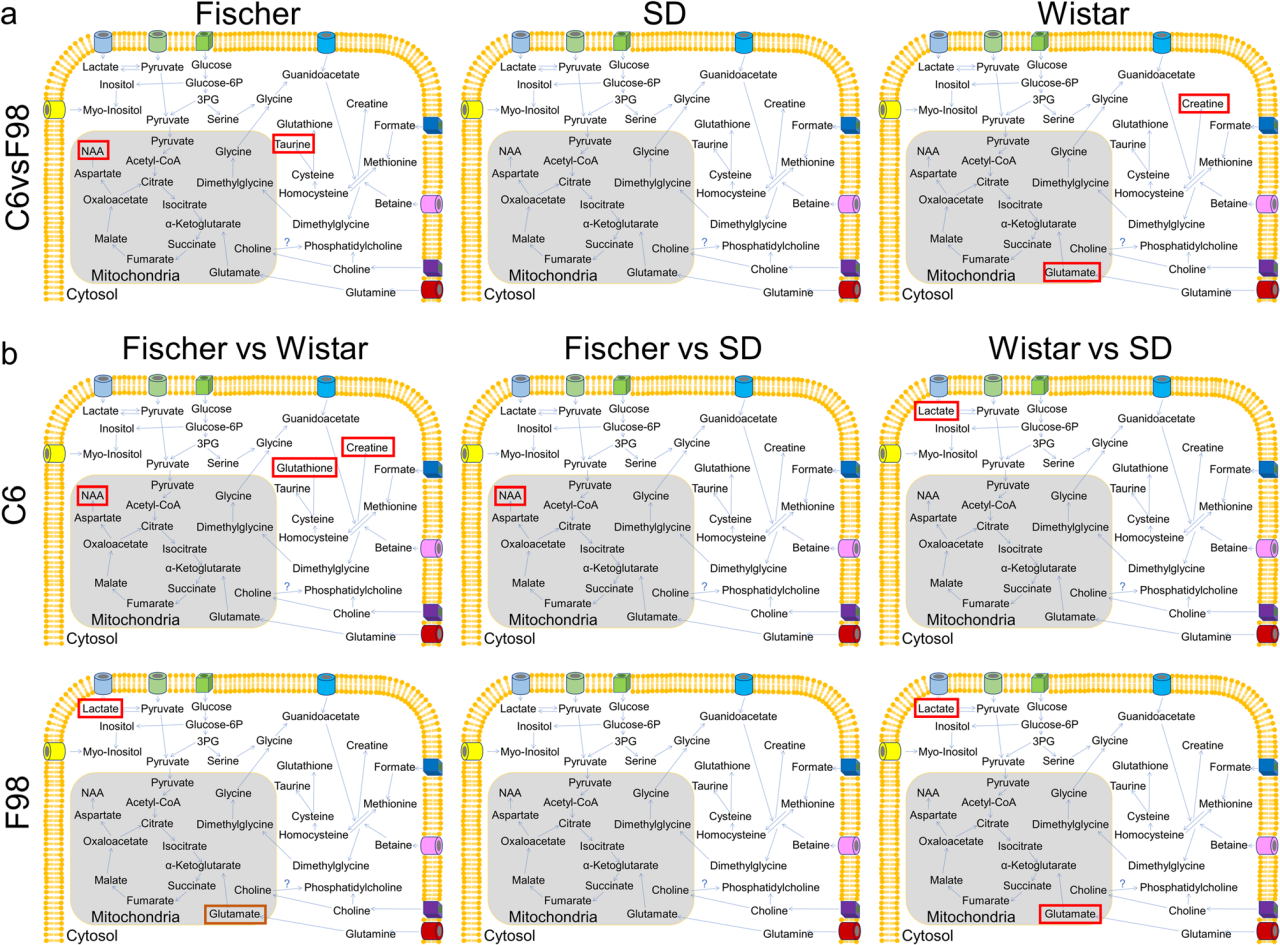
Scheme comprising significant components of the local metabolism in glioblastoma models. (**a**) Comparison between different glioblastoma cells implanted in different strains, and (**b**) comparison between the same cell line implanted in different strains. In this comparison, orange rectangles indicate statistical trends (*p* < 0.1), and red rectangles indicate statistical significance differences (*p* < 0.05).

Comparisons between different glioblastoma cells implanted in the same strain or the same cell line in different strains revealed several differences in metabolite profiles. When comparing C6-bearing rats with F98-bearing rats, significant variations were observed in NAA and Tau levels in Fischer rats, while Wistar rats showed significant differences in tCr and Glu levels (Fig. 6a). However, no significant changes were detected in SD rats (Fig. 6a). Conversely, analyzing different strains implanted with C6 cells revealed significant variations in glutathione, NAA, and tCr between Fischer and Wistar rats, and a trend in NAA compared to SD rats (Fig. 6b). Moreover, significant variations in lactate were detected when comparing Wistar and SD rats (Fig. 6b). Finally, for rats implanted with F98 cells, few changes were distinguished, mainly variations in lactate when comparing Wistar rats with Fischer rats or SD rats (Fig. 6b) and in Glu, being statistically significant for Wistar vs. SD rats, and a trend between Wistar and Fischer rats.

### Survival rate

**Fig. 7:**
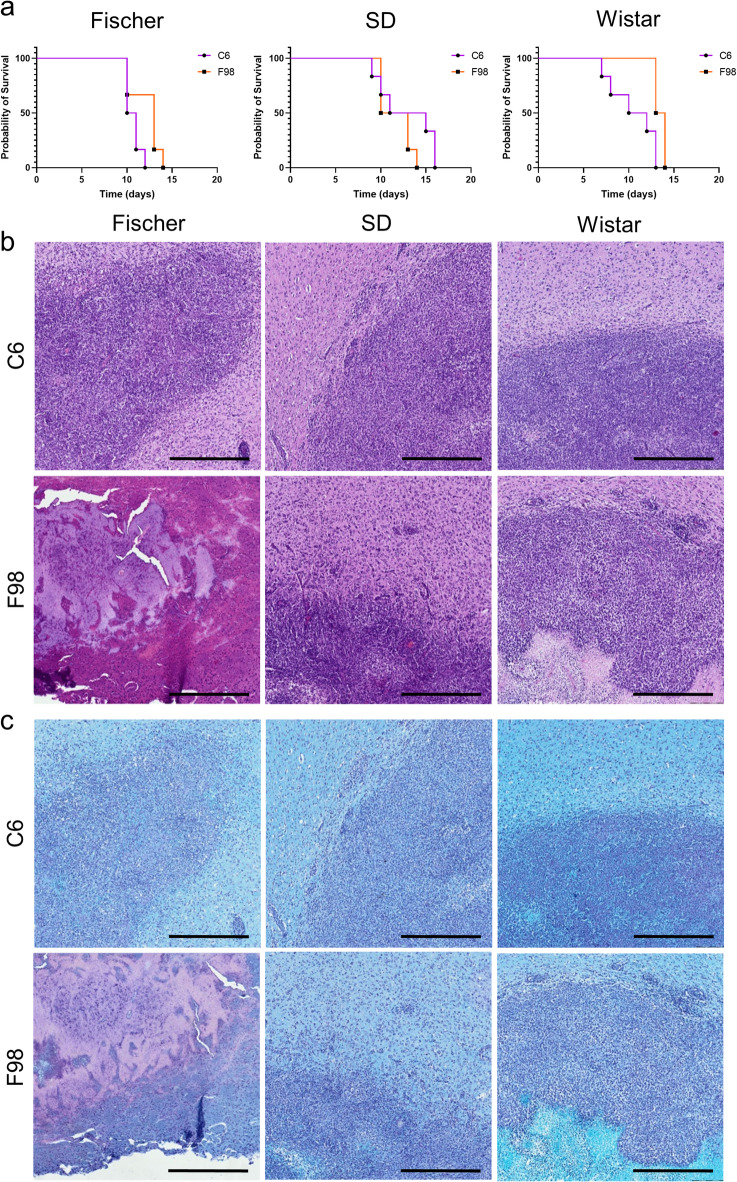
(**a**) Kaplan–Meier survival plots for glioblastoma-bearing rats (n = 8). The survival times in days after tumor implantation have been plotted for two different types of cells, C6 and F98, denoted by (■) and (○), respectively. Additionally, histological slices of rat brains at their endpoints are stained with H&E (**b**) LFB (**c**). Tumor cells are stained purple with H&E, and white matter tracks are stained blue with LFB. The strains of rats used in the study, Fischer, SD, and Wistar, were placed from left to right, respectively. The scale bar corresponds to 500 µm.

Survival time was estimated using Kaplan–Meier survival curves (Fig. 7a). Significant differences (*p* < 0.05) in survival were observed in Fischer and Wistar rats implanted with different cell lines, but not in SD rats. Notably, variations depended on the rat strain, with significant differences observed at the initiation or conclusion of the experiment. Fischer rats began to exhibit mortality around day 10, irrespective of the implanted cells. Additionally, F98-Fischer showed a significant delay in mortality (2 days later) compared to C6-Fischer. Conversely, C6-Wistar rats experienced earlier mortality compared to F98-Wistar, but the experiment concluded on days 13–14 after tumor implantation, regardless of the cell line. Notably, 40% of C6-Fischer rats did not develop glioblastoma.

### Ex vivo analysis

Histological sections of tumor-bearing rat brains stained with H&E and LFB provided valuable insights into tumor cytoarchitecture and invasiveness (Fig. 7b–c). In H&E staining, tumor cells appeared purple, contrasting with the pink-stained normal brain parenchyma, while in LFB staining, they appeared purple against the blue-stained white matter tracks of healthy brain tissue, thereby facilitating accurate evaluation of tumor infiltration. Both C6 and F98-derived glioblastomas displayed high cellularity and dense cell packing compared to normal brain parenchyma. Notably, in C6-SD and all F98-derived glioblastomas, cells migrated extensively from the tumor periphery, resulting in irregular tumor margins. In contrast, C6-Fischer and C6-Wistar displayed continuous tumor margins. Additionally, the bright blue myelin stain was unevenly distributed in the tissue surrounding C6-SD tumors and all F98-derived glioblastomas, indicating a more infiltrative growth pattern.

## Discussion

Animal models play a crucial role in cancer research, particularly in elucidating the complexity of tumor physiopathology and evaluating new therapeutic strategies. Choosing the most appropriate model is essential, as it can significantly streamline the translation from preclinical to clinical research, saving valuable time. Magnetic resonance techniques serve as valuable tools in guiding this selection process, offering insights into imaging and metabolomic features to identify the animal models that more closely resemble human glioblastoma. The blood–brain barrier (BBB) is a characteristic feature of normal brain vascularization, yet high-grade gliomas typically exhibit vascular pathology and BBB dysfunction^34^, leading to the extravasation of intravenously injected MRI CAs, particularly gadolinium chelates. The typical enhancement pattern of human glioblastoma lesions evaluated by DCE-MRI entails a quick and intense uptake at the tumor periphery, corresponding to well-vascularized proliferative regions. Conversely, a slower uptake at the tumor center is usually observed, reflecting necrotic areas. Following a brief period (a few minutes), the contrast agent begins to be washed out from the proliferative regions, confirming the high vascularization in those areas^35^. The CA extravasation behavior in most of the glioblastoma ORMs described in this study mirrors that observed in human patients. However, exceptions were noticed, particularly in the F98-Wistar and C6-Fischer groups, where both the wash-in and wash-out slopes exhibited significant differences compared to the other groups and, consequently, to human glioblastomas. Additionally, tumor K_trans_ has been previously associated with the pathological grade of gliomas, with values ranging from 0.01 to 0.04 min^−1^ for grade I and from 0.07 to 0.21 min^−1^ for grade IV^36,37^. Therefore, the K_trans_ values determined for all our ORMs, ranging from 0.12 to 0.24 min^−1^, are well-aligned with those reported for grade IV human gliomas. This finding has significant implications for the use of these models in studies where mimicking human glioblastoma vascular permeability is essential. This is particularly relevant in the rapidly growing field of nanotechnology, where numerous diagnostic and therapeutic nanosystems are continuously being reported, many of which are designed for passive tumor targeting by leveraging the leakiness of tumor vasculature^38,39^. Furthermore, V_e_, which represents the volume of the extravascular extracellular space (EES) per unit volume of tissue in which the CAs can be accumulated, has also been associated with glioma grade, with reported values ranging from 0.35 to 0.87 for grade IV gliomas^36,37,40^. Our values, ranging from 0.28 to 0.61, are mostly within the range of human glioblastomas, with the higher values observed in F98-Fischer, C6-Wistar, and C6-Fischer. Additionally, the peritumoral area showed lower values than the tumor periphery and higher than the healthy brain, which is also in agreement with reported values for human gliomas^41^.

The evaluation of T_2_ provides valuable information about the tumor extent and insights into factors such as tumor progression^42^ or invasiveness^43,44^. Increased T_2_ values indicate higher water content within a tissue, while MD provides information on the motion restriction of the water molecules^28^. Therefore, both parameters are essential for evaluating certain tumor features, such as the appearance of surrounding edema. Interestingly, in ORMs implanted with F98 cells but not with C6, the peritumoral area showed higher T_2_ values than the tumor periphery, which, alongside increased MD values, denoted the presence of vasogenic edema. Vasogenic edema is commonly observed in human glioblastoma patients and is associated with poor prognosis^45^. Other authors have also noticed this differential behavior between C6 and F98 [13], suggesting that F98 may more closely resemble human glioblastoma. Indeed, peritumoral MD values around 1670 µm^2^/s have been reported in human glioblastomas^46,47^, which are in good agreement with our data for F98 models, with values ranging from 1549 to 1807 µm^2^/s, the latter corresponding to the original F98-Fischer model. In contrast, all C6 models showed significantly lower values, ranging from 1167 to 1336 µm^2^/s, with the lowest value corresponding to C6-Wistar. Concerning tumor MD values, they are lower in high-grade human gliomas compared to low-grade, with mean MD varying from 800 to 1200 µm^2^/s^48^. Additionally, a minimum MD value of 980 µm^2^/s seems to be the optimal threshold for differentiation between low and high-grade gliomas. However, our ORMs displayed tumor MD values ranging from 1031 to 1531 µm^2^/s, which are slightly higher than those of human glioblastomas. Strikingly, C6-SD and F98-SD models showed the lowest values, suggesting that both cell lines grow with cellular density closer to human glioblastomas when implanted in SD animals.

In addition to MD, other interesting parameters can be extracted from the DTI experiments, such as FA, which provides information directly related to tissue microstructure, with a decrease in FA being associated with the destruction of nerve fibers. Our analysis revealed FA values below 0.25 in the tumor periphery of all our ORMs, which are well aligned with those reported for human glioblastomas^49^. Regarding the peritumoral area, FA values were lower in F98 models, with the lowest value corresponding to F98-Fischer. These findings are consistent with the more infiltrative growth pattern of F98 tumors observed in the histological analysis. Although diffusion kurtosis imaging (DKI) can provide more detailed insight into tissue microstructure at high b‑values, we chose standard DTI for its wide validation and ease of use in preclinical settings. Our two‑shell protocol (b = 400 and 1800s/mm^2^) offers a practical balance between sensitivity to microstructural changes and stable tensor fitting, delivering reliable FA and MD values without the extra complexity or potential instability of higher‑order models. Nevertheless, DKI will be considered in future studies as it could provide a more comprehensive analysis of the microstructural heterogeneity of glioblastoma.

Finally, magnetization transfer studies provide insight into the macromolecular microenvironment of the tissue, with proliferative tumor areas exhibiting higher MTR values than necrotic parts or regions with vasogenic edema. The average MTR of human glioblastomas has been calculated to be ≈ 25%^50^, matching the value obtained for the tumor periphery of all ORMs.

In summary, the F98 models are the most similar to human glioblastomas in terms of MRI parameters, exhibiting comparable values of vascular permeability and extracellular volume fraction, the presence of peritumoral edema, tumor macromolecular microenvironment, and infiltrative growth, regardless of the rat strain in which they are implanted. However, the CA wash-in and wash-out slopes were only comparable to human glioblastomas in the case of Fischer and SD but not in Wistar rats. Overall, no significant differences in tumor growth, as evaluated by multiparametric MRI, were observed for F98-Fischer and F98-SD, despite the former being a syngeneic model, while the latter is clearly allogenic. As for C6 tumors, they differ more from human glioblastomas, typically lacking peritumoral edema and exhibiting a less infiltrative pattern. Moreover, C6 tumors were similar in Wistar and SD rats, while in Fischer rats, only 60% of the animals developed tumors (vs. 100% in the other two strains) and showed differences in CA wash-in and wash-out slopes.

Metabolic reprogramming is a well-recognized hallmark of cancer, playing a crucial role in driving tumor cell transformation and progression^51^. The local metabolism of human glioblastomas evaluated by MRS is characterized by drastically decreased concentrations of NAA, decreased concentrations of mI and tCr, and increased concentrations of Gln, tCho, Gly, and lactate, alongside the presence of a prominent signal from mobile lipids^52–54^.

NAA is considered a neuronal marker as it is predominantly located in neurons^55^. NAA levels are, therefore, drastically decreased in glioma tumors, showing a correlation with glioma grading^56^. Surprisingly, although NAA values were lower in tumors than in the contralateral tissue, only the C6-Fischer and C6-SD models showed statistically significant decreases. This result could be explained, at least in part, by chemical shift displacement errors (CSDE) in in vivo MRS, which are more prominent at high magnetic fields, leading to the contribution of healthy parenchyma to the overall voxel signal^57^. Interestingly, though, NAA levels of the contralateral “healthy” tissue tend to be lower in F98 models than in C6 models, which is likely related to the more infiltrative growth pattern of F98 tumors.

Inositol and glycine largely overlap in vivo, making it challenging to quantify both metabolites separately^58^. However, due to the complex J-coupling pattern of mI methine protons, at relatively long echo times, such as 144 ms, the mI signal significantly decreases, and the Gly signal becomes predominant and thereby distinguishable from mI^52^. mI is a sugar-like molecule primarily located in glial cells, serving as an osmolyte and participating in membrane turnover^59^. mI levels significantly increase in low-grade gliomas due to low cell dedifferentiation, while they decrease drastically in high-grade gliomas^60^. On the other hand, elevated levels of Gly have been associated with increased cell proliferation and aggressiveness in human gliomas, where reprogramming glycine-mediated one-carbon metabolism seems to be one of the mechanisms to meet the biosynthetic demands for the rapid cell proliferation of these tumors^61^. Notably, the mI + Gly levels are elevated in all of our ORMs, although only F98-Fischer and C6-Wistar show significant differences with respect to the contralateral hemisphere. Long TE spectra indicate that this signal increase is attributable to Gly. Therefore, both models display this characteristic hallmark of aggressive gliomas, although with higher values in C6-Wistar.

Decreased tCr levels have also been reported as a metabolic hallmark of high-grade gliomas^62^. The increased consumption of phosphocreatine for ATP production, combined with changes in cellular metabolism, results in decreased tumor tCr levels. Although lower levels of tumor tCr compared to contralateral brain are found in all ORMs, statistical significance was unexpectedly detected exclusively in the F98-Wistar, C6-Fischer, and C6-SD models. Indeed, these decreases were much less pronounced than those described in humans, where an approximately threefold decrease in tCr was noted in tumor tissue compared to normal parietal white matter^53^, whereas our ORMs showed a 1.6-fold decrease in tumor tCr, with a minimum tCr tumor value of 4.5 ± 1.2 mM for C6-Fischer. Therefore, none of the ORMs resemble the creatine metabolism observed in human glioblastoma.

Regarding Gln, due to the high metabolic demand of brain tumors, they act as ‘glutamine traps’, effectively competing for the Gln that is recycled by astrocytes^63^. This phenomenon can explain the higher levels of Gln observed in glioblastomas^64^. Although several ORMs exhibited increased tumor Gln levels, these increases were only statistically significant in the C6-Wistar model and with a statistical trend in the F98-SD model.

Alterations of choline metabolism in tumors have been known since the pioneering work of Griffiths and coworkers 4 decades ago, showing increased levels of phosphomonoesters in tumor tissue by in vivo ^31^P MRS^65^. Since then, countless studies have demonstrated that disrupted choline metabolism is a hallmark of cancer, being associated with progression and aggressiveness^66,67^. Cancer cells require higher levels of choline derivatives for membrane formation. Moreover, altered signaling pathways boost choline uptake and use. These changes contribute to cancer development, progression, and resistance to treatment, driven by both elevated choline needs and alterations in metabolic enzyme activity^68,69^. Our results are in good agreement with the reported findings in human glioblastoma, as all our ORMs exhibited statistically significant increases in tCho. Notably, tumor tCho levels were similar for both cell lines, irrespective of the rat strain in which they were implanted.

Another interesting phenomenon occurring in glioblastoma metabolism is the well-known Warburg effect^70^, which leads to increased lactate levels within the tumor. Three of our ORMs, F98-Fischer, F98-SD, and C6-SD, showed significantly increased levels of lactate in the tumor tissue. Notably, lactate levels in the F98-Fischer model were the closest to those reported in human glioblastomas, with concentrations of 11.4 ± 4.3 mM for the F98-Fischer model and 11.7 ± 7.0 mM for human glioblastoma.

As for the correlation matrix, the high SCC among contralateral samples across the different models suggests a strong homogeneity within these samples. Similarly, the F98-Fischer and C6-Wistar models display high homogeneity among individuals within each group, reflecting the stability of these models. In contrast, the Wistar-F98 and Fischer-C6 models turned out to be the most heterogeneous. Interestingly, the greatest metabolic differences between tumors and contralateral regions were observed in the SD models, which showed the lowest SCC values.

Overall, among the ORMs studied here, the metabolic profiles of F98-Fischer and C6-Wistar are the closest to human glioblastomas, exhibiting some of the most relevant metabolic hallmarks of these tumors, yet not exactly the same. F98-Fischer shows elevated levels of Gly, tCho, and lactate, whereas C6-Wistar exhibits elevated levels of Gly (the highest), tCho, and Gln. Additionally, both metabolic profiles are homogeneous among individuals, supporting the higher stability of these two models. However, they also show some important metabolic discrepancies with respect to human glioblastomas, namely moderately decreased levels of NAA and tCr, which contrast with the drastic decreases observed in patients.

In this context, it is worth noting that absolute metabolite quantification in vivo is highly challenging due to the presence of multiple sources of error that must be accounted for to obtain actual absolute concentrations, even when using the unsuppressed water signal as an internal reference. These sources include variations in tissue water content, differences in relaxation times (T_1_ and T_2_) of both metabolites and water, and partial volume effects arising from the inclusion of multiple tissue types within the voxel^71,72^. In the normal brain of a homogeneous population, as is often the case in preclinical animal studies, these differences can be minimized by carefully replicating voxel positioning across subjects^73^. However, in tumor tissue, particularly in glioblastomas, variations in water content and relaxation times have been shown to lead to an underestimation of metabolite concentrations compared to healthy brain tissue^74^. Therefore, our findings regarding metabolites that appear to be decreased in tumors relative to the healthy brain tissue, namely NAA and tCr, should be interpreted with caution. In contrast, for metabolites that are significantly increased in tumor tissue, such as tCho, Gly, lactate, and Gln, we can be confident that these differences are genuine and likely even larger than observed. On the other hand, it is also important to note that our metabolite estimates, although not corrected for differences in water content or relaxation times in the tumor tissue, are directly comparable to those reported in several relevant human MRS studies that also reported uncorrected data^52,53,58^.

Finally, the histological evaluation confirmed the MRI findings, demonstrating that all F98 tumors exhibit an infiltrative growth pattern, regardless of the rat strain in which they are implanted, while C6 tumors show well-defined tumor margins, consistent with previous studies^75^. An exception was noted for C6-SD, which, similarly to F98, is more infiltrative. Therefore, the histological evaluation further supports the closer similarity of F98 tumors to human glioblastoma, characterized by their diffuse infiltrative growth^76,77^. Furthermore, the high tumor cell density observed in H&E-stained sections is consistent with lower MD values in the tumor periphery when compared to the adjacent parenchyma, reinforcing the concept that increased cellularity restricts water difusión, a hallmark of high-grade gliomas.

Our results indicate that, among ORMs studied, the F98-Fischer most closely resembles human glioblastomas, exhibiting similar infiltrative growth, vascular permeability, and key metabolic hallmarks. In contrast, the C6-Wistar model, while also displaying notable similarities in vascular permeability and various critical metabolites, does not replicate the infiltrative growth pattern observed in human tumors. Nevertheless, both models share significant similarities with human glioblastomas, making them valuable options for preclinical research. However, carefully considering their discrepancies is crucial for selecting appropriate models based on the specific research question. For instance, the F98-Fischer model may be preferred for studies on the mechanisms of tumor invasion, while both models can be effectively used for research focused on vascular permeability.

Furthermore, this study reveals a complex interaction with the host microenvironment. While the F98 cell line consistently displayed an infiltrative growth pattern regardless of the rat strain, indicating a stronger influence of its inherent characteristics, the C6 cell line showed more pronounced variations depending on the rat strain. Thus, the growth pattern of C6 tumors shifted from well-defined margins in Wistar and Fischer rats to more infiltrative growth in SD rats, suggesting that the host microenvironment plays a more significant role in shaping the C6 tumor phenotype. Regarding metabolism, although certain variability across different strains was observed for both cell lines, these differences were more pronounced in C6, further supporting the stronger effect of the host on the C6 tumor compared to F98.

These were unexpected findings, as the shift from a syngeneic model (F98-Fischer) to allogeneic models (F98-Wistar and F98-SD) was anticipated to produce more pronounced changes in F98 compared to C6, given that the original C6-Wistar model was already allogeneic and characterized for eliciting a strong immunogenic response. This highlights the complexity of the interplay between tumor genetics and the host microenvironment, emphasizing the need to consider both factors when selecting the most appropriate model to address specific research questions.

Future studies incorporating immunohistochemical analyses, such as evaluating tumor-associated macrophage (TAM) infiltration, may help elucidate the heterogeneity of the immune response across different glioblastoma models and provide further insights into tumor-host interactions.

## Conclusions

This study highlights the power of multiparametric magnetic resonance imaging and spectroscopy in the characterization of tumor models, providing insights into fundamental tumor features, such as the presence of necrosis or edema, infiltration into the surrounding tissue, vascular permeability, cell density, or metabolism, among others.

Using these advanced techniques for the in vivo characterization of F98 and C6 glioblastoma models, the F98-Fischer model emerged as the closest to human glioblastomas, with strong similarities in vascular permeability, infiltrative growth pattern, and key metabolic hallmarks. The C6-Wistar model also showed notable similarities, particularly in vascular permeability and metabolic profile, but lacked the infiltrative growth pattern. Consequently, the F98-Fischer model would be particularly advantageous for studies on glioblastoma infiltration, while both the F98-Fischer and C6-Wistar models would be valid options for other studies, such as those focused on vascular permeability.

Notably, although both models exhibited key metabolic hallmarks of human glioblastomas, they also showed some discrepancies, particularly higher-than-expected levels of NAA and tCr, stressing the need for careful consideration when using these models for studies focused on tumor metabolism.

Furthermore, the evaluation of host-tumor interaction by implanting both cell lines in different rat strains showed that the F98 cell line consistently produced infiltrative tumors across all rat strains despite the transition from a syngenic F98-Fischer model to allogenic models in the other strains, suggesting a predominant effect of its genetic background on the tumor phenotype over the host microenvironment. In contrast, the C6 cell line showed more variability depending on the rat strain, indicating a greater susceptibility to the host microenvironment despite its allogenic origin. This finding underscores the complex interplay between tumor genetics and host factors in shaping tumor phenotype and emphasizes the importance of considering host-tumor interactions when selecting models for glioblastoma research.

## Electronic supplementary material

Below is the link to the electronic supplementary material.

Supplementary Material 1

## Data Availability

The datasets used and/or analyzed during the current study are available from the corresponding author upon reasonable request.

## Abbreviations

CA: Contrast agent
CNS: Central nervous system
DCE: Dynamic contrast enhancement
DTI: Diffusion tensor imaging
DWI: Diffusion-weighted imaging
FA: Fractional anisotropy
Gly: Glycine
Glu: Glutamate
Gln: Glutamine
H&E: Haematoxylin and eosin
LFB: Luxol fast blue
MD: Mean diffusivity
mI: Myo-inositol
MRI: Magnetic resonance imaging
MRS: Magnetic resonance spectroscopy
MTR: Magnetic transfer ratio
NAA: *N*-acetyl aspartate
ORMs: Orthotopic rat tumor models
PWI: Perfusion-weighted imaging
RCE: Relative contrast enhancement
SCC: Spearman correlation coefficient
SD: Sprague Dawley
Tau: Taurine
tCho: Total choline
tCr: Total creatine
TCA: Tricarboxylic acid
WHO: World Health Organization
