# Supplementary material for: A multiparametric perspective on C6 and F98 cell lines in orthotopic rat models for glioblastoma research
Source: Sci Rep. 2025 Jul 2;15:22547. doi: 10.1038/s41598-025-06684-5 (PMC12214515; doi:10.1038/s41598-025-06684-5)
Supplement: Supplementary file 1 — Supplementary Material 1 [file 41598_2025_6684_MOESM1_ESM.pdf]

Supplementary Information

A multiparametric perspective on C6 and F98 cell lines in orthotopic rat models for glioblastoma research

Carlos Caro[1,2,†], Nuria Arias-Ramos[3,†], Jesús David Urbano-Gámez[1,2], Raquel González-Alday[3], Pilar López-Larrubia[3,4,5], María Luisa García-Martín[1,2,6,*].*

[1]Biomedical Magnetic Resonance Laboratory-BMRL, Andalusian Public Foundation Progress and Health-FPS, Seville, Spain

[2]Instituto de Investigación Biomédica de Málaga y Plataforma en Nanomedicina - IBIMA Plataforma BIONAND, C/ Severo Ochoa, 35, 29590 Málaga, Spain

[3]Instituto de Investigaciones Biomédicas Sols-Morreale (CSIC-UAM). Arturo Duperier, 4, 28029 Madrid, Spain.

[4]Biomedical Research Networking Centre On Rare Diseases (CIBERER), Institute of Health Carlos III, Madrid

[5]Instituto de Investigación Sanitaria La Paz (IdiPaz), Madrid.

[6]Biomedical Research Networking Center in Bioengineering, Biomaterials & Nanomedicine (CIBER-BBN), 28029 Madrid, Spain

†Both authors contributed equally to this work

* email: plopez@iib.uam.es / mlgarcia@ibima.eu

Index

1. Methods

A) Cell culture

B) Magnetic Resonance (MR) studies

C) Histology

2. Results

A) Anatomical MRI and Dynamic Contrast Enhancement (DCE)

B) Diffusion Tensor Imaging (DTI)

C) Magnetic Transfer Ratio (MTR)

D) ^{1}H NMR Spectra

1. METHODS

A) Cell culture. Both C6 and F98 rat GBM cells were cultured using DMEM as the growth medium (supplemented with L-glutamine (2 mM), FBS (10%), and penicillin/streptomycin (1%)). Both cell lines were cultured at 37 ºC in an incubator (5% CO_2).

B) Magnetic Resonance (MR) studies. Animals were anesthetized by inhalation of oxygen (1 L/min) containing 3% to 4% of isofluorane in an induction box, and then, anesthesia was maintained with a flow of 1% to 1.5% of isofluorane by employing a nose mask during the experiments. The animals were placed in a probe with a recirculating water system, which maintained the body temperature at approximately 37 ºC. The physiological monitoring of the animals was performed by a gating system designed for small animals (SA Instruments, Inc., Stony Brook, NY, USA), controlling the body temperature and respiratory rate.

Magnetic Resonance Imaging (MRI)

Anatomical images: High-resolution T_2-weighted images (T_2WI) covering the entire tumor in axial, sagittal, and coronal orientations were acquired every 2-3 days to follow up the tumor development using a turbo-rapid acquisition with relaxation enhancement (RARE) sequence with TR = 2500 ms, TE = 33 ms, Number of averages (Av)= 2, RARE factor= 8, Acquisition time (AT)= 4 min, Field of View (FOV)= 3.2 cm^2, matrix= 384x384 pixels (corresponding to an in-plane resolution of 83.33 μm^2) and 1 mm slice thickness (no inter-slice gap). Tumor volumes were calculated by summing the volumes in each individual slice based on the T_2WI.

Multiparametric-MRI: When tumors presented a size of approximately 1.5 cm^3, a multiparametric MRI study was performed. All the images in this study were acquired with the same axial orientation geometry parameters: 5 slices with 1 mm slice thickness covering the most part of the tumor, a Field of View (FOV)= 3.2 cm^2 and an acquisition matrix=192 x 192 pixels (corresponding to in-plane resolution of 166.7 μm^2). The following images were acquired:

- T_2 maps were acquired using a multi-slice multi-echo (MSME) sequence with TR= 3000 ms, TE= 7 ms, Number of echoes = 32, Av= 1 and AT= 7 min, 12 s.
- Magnetization Transfer Imaging was calculated by acquiring two sets of RARE images with the following parameters: TR= 2500, TE= 11 ms, Av= 1, RARE factor= 2, AT= 4 min. One of the images was acquired with a magnetization transfer (MT) pulse, which employed 50 radiofrequency pulses of 10 µT with a bandwidth= 1500 Hz, length= 1.82 ms, and offset= 2000 Hz.
- Diffusion Tensor Imaging: A Stejskal-Tanner sequence with an echo-planar (EPI), where TR = 2500 ms, TE = 33 ms, Av =3, 4 segments, diffusion gradient separation (Δ) = 20 ms, diffusion gradient duration (δ) = 4 ms, one basal image (without diffusion gradient application) and two b factors of 400 and 1800 s/mm^2, corresponding to a diffusion gradient strength of 31.3 % and 66.4%, respectively, applied in seven directions. AT= 7 min, 30 s.

- T_1 map was acquired using a RARE at variable TR (RARE-VTR) sequence with variable TR with 7 TR values (TR=100, 200, 400, 800, 1500, 3000, 8000 ms), TE= 6.4 ms, Av= 1, Flip angle (FA)= 90°, AT= 16 min, 48 s.
- Dynamic Contrast Enhancement (DCE): A Fast low angle shot (FLASH) was used with TR= 27.8 ms, TE= 2.5 ms, Av= 1, Number of repetitions= 240, FA= 12°. AT= 16 min. A manual intravenous injection of Gadovist (0.3 mmol/kg, 300 µl) was administered 1 min after the beginning of the acquisition (corresponding to 15 images), over a period of ≈ 2 seconds.

Magnetic Resonance Spectroscopy (MRS)

Single voxel spectra were acquired in the tumor and in the contralateral healthy brain area with a voxel volume of 4x4x4 mm^3 using a Point REsolved Spectroscopy (PRESS) sequence with TR= 3000 ms, TE= 35 ms or 144 ms, 128 averages, a spectral width of 4401.41 Hz, and 2048 data points. Water suppression was performed using VAPOR (Variable Power RF Pulses with Optimized Relaxation Delays). A non-suppressed water spectrum was acquired with 4 averages to be used as a reference for metabolite quantification. Localized shimming was performed using field mapping up to third-order shims, as implemented in ParaVision 6.1. software (Bruker BioSpin GmbH, Ettlingen, Germany).

MRI and MRS processing and analysis

One central slice covering the largest part of the tumor of from the parametric maps obtained of each rat was processed with in-house developed software (My Map Analyzer) built in MatLab (MathWorks, Natick, MA, USA)[1,2] in which color-based maps were generated by pixel-wise fitting the signal intensities to the appropriate equation:

- T_2 maps were calculated by equation 1, where S is the signal intensity of a pixel in the image for a specified TE, and S_0 is the signal intensity of the same pixel at TE= 0:

$$S = S_0 . e^{-TE/T2}$$

- The Magnetization Transfer Ratio (MTR) effect was calculated according to equation 2, where SMT is the signal intensity of a pixel in the image with the application of the MT pulses, and S0 is the signal of the same pixel without it:

$$MTR = \frac{S_0 - S_{MT}}{S_0} \; x \; 100$$

- The DTI parameters, mean diffusivity (MD), and fractional anisotropy (FA) maps were calculated according to equations 3 and 4, where the corresponding eigenvalues (λ_1, λ_2, λ_3) were obtained by solving the tensor.

$$MD = \frac{\lambda_1 + \lambda_2 + \lambda_3}{3}$$

$$FA = \frac{\sqrt{(\lambda_1 - MD)^2 + (\lambda_2 - MD)^2 + (\lambda_3 - MD)^2}}{2(\lambda_1^2 + \lambda_2^2 + \lambda_3^2)}$$

- T_1 maps were calculated by Equation X, where S is the signal intensity of a pixel in the image for a specified TR, and S_0 is the signal intensity of the same pixel at TR= 0:

$$S = S_0.(1 - S_0.e^{-TR/T1})$$

Two regions of interest (ROIs), tumor periphery and peritumoral area, were manually selected from parametric maps and quantified with ImageJ (National Institutes of Health, Bethesda, MD, USA, http://rsbweb.nih.gov/ij/).

- DCE data from the same central slices used for the rest of the maps were processed with DCE@urLab software[3] using the Reference Region Model.[4] The volume transfer constant between plasma and tumor extravascular-extracellular space (Ktrans) and the extravascular-extracellular space volume fraction (Ve) were obtained. Both parameters were calculated for the tumor periphery and peritumoral area, using a head-muscle region as reference. Relative contrast enhancement (RCE) curves were also obtained with this software, calculating the percentage of MRI signal obtained at each time point relative to the mean MRI signal obtained at the baseline. The RCE curves were measured in three different regions: the tumor periphery, peritumoral area, and contralateral parenchyma (equivalent, as much as possible, in size and location to the tumor mass).

- MRS were processed using LCModel (ver. 6.3, S. Provencher, Oakville, Canada),[5] where ^1H NMR spectra are fitted to a metabolite database provided by the software developer, generated explicitly for Bruker PRESS spectra acquired at 9.4 T with our acquisition conditions. Only metabolite concentrations with standard deviations below 20% were considered for statistical analysis. Technical outliers, which refer to spectra exhibiting broadened peaks or distorted baselines due to poor shimming and/or inefficient water suppression, were discarded from the analysis.

C) Histology

Animals were euthanized by decapitation using a guillotine. Then, tissue samples were collected and fixed in 4% formaldehyde (Panreac, pH 7 buffered) for 48 h, changing the 4% formaldehyde after 24 h. Then, the samples were dehydrated through graded ethanol and embedded in paraffin (temperature 56° C for 2 h under stirring and vacuum) using a Leica TP1020 histoprocessor. The detailed procedures are described below.

Haematoxylin and Eosin (H&E): paraffin-embedded samples were sectioned at 7 µm thickness, then deparaffinized, rehydrated, and stained with H&E, and then dehydrated in ascending concentrations of ethanol, cleared in xylene, and mounted on commercial glass slides.

Luxol Fast Blue (LFB): paraffin-embedded samples were sectioned at 7 µm thickness, then deparaffinized, rehydrated, stained with Luxol Fast Blue and then dehydrated in ascending concentrations of ethanol, cleared in xylene, and mounted on commercial glass slides.

2. RESULTS

A) Anatomical MRI and Dynamic Contrast Enhancement (DCE)

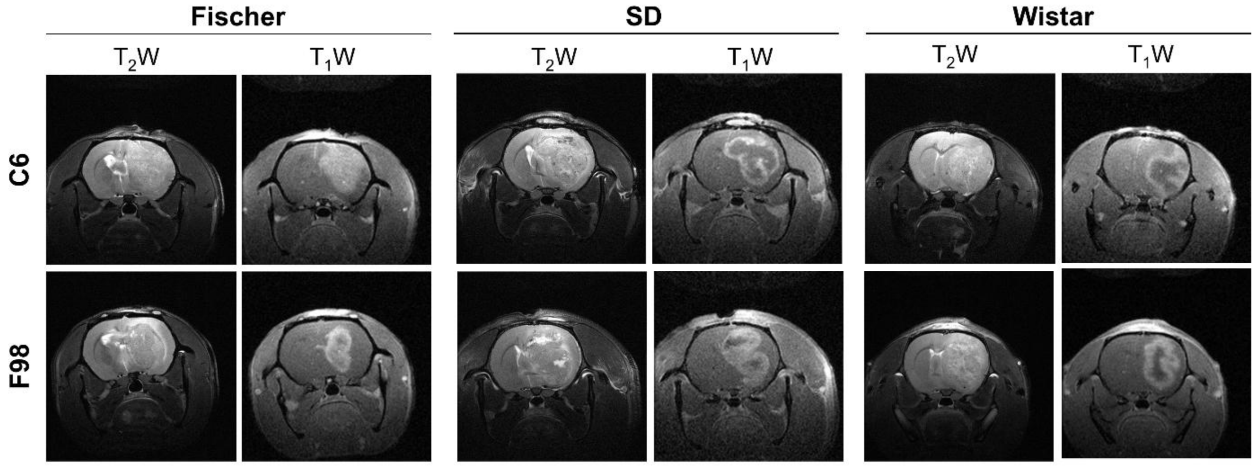

Figure S1. T_2-weighted (T_2W) images before and T_1-weighted (T_1W) images after the intravenous CA administration of the different ORM studied.

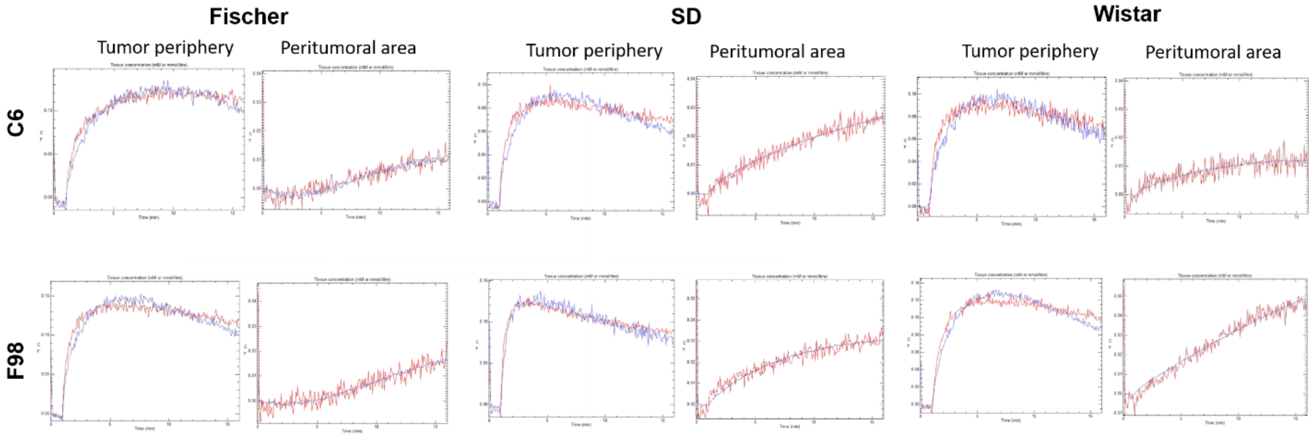

Figure S2. Representative ROI fitting examples using the RR model for each study group, obtained from the DCE@OURLAB software. The blue curve represents the observed signal in the region of interest, while the red curve shows the fitted model.

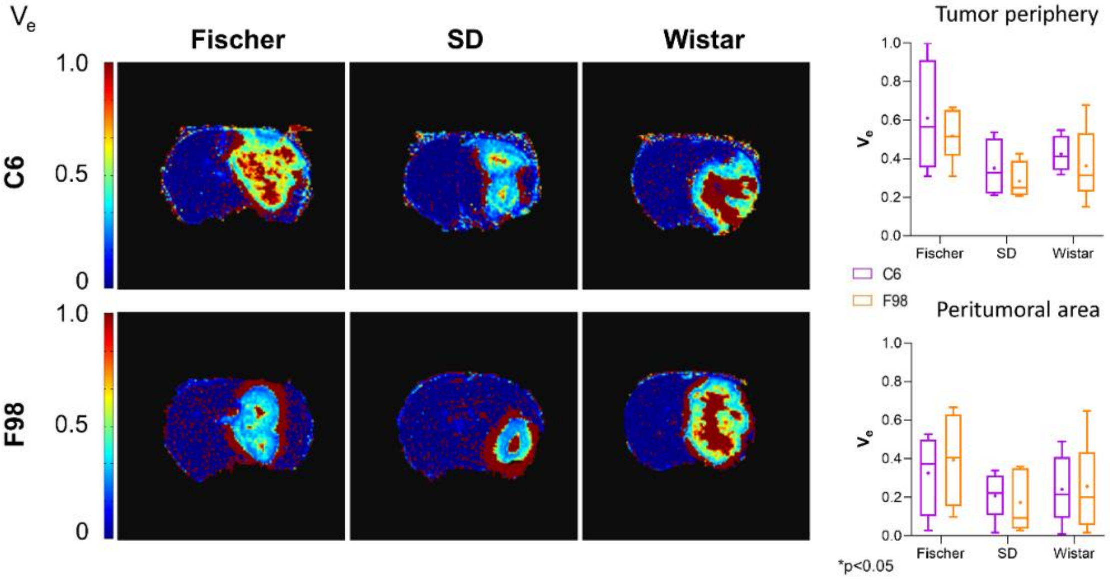

Figure S3. V_e maps analysis. On the left, V_e maps obtained from DCE images of a representative rat from each ORM. On the right, tumor periphery and peritumoral area V_e quantification of the different models represented as boxplots.

B) Diffusion Tensor Imaging (DTI)

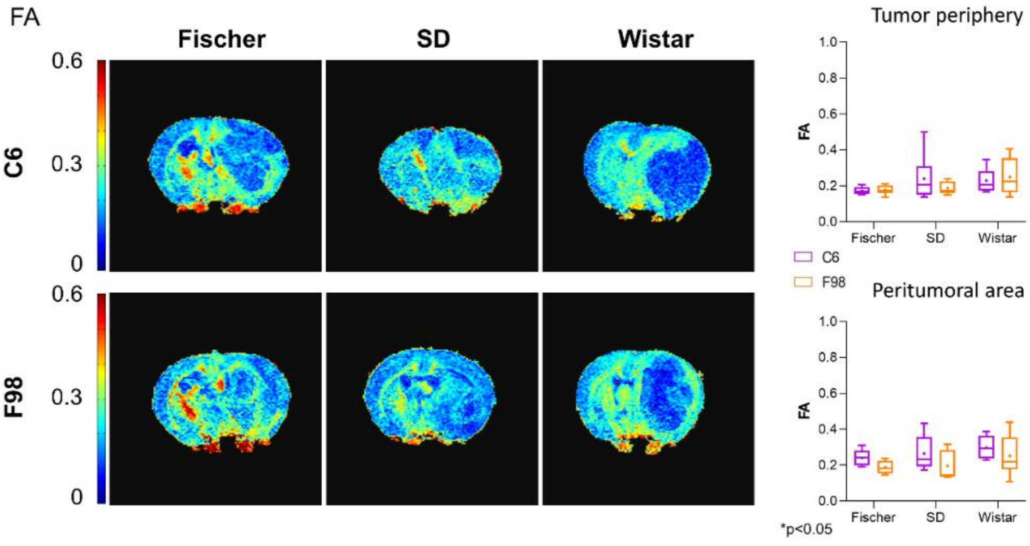

Figure S4. FA maps analysis. Left: FA maps obtained from DTI images of a representative rat from each ORM. Right: tumor periphery and peritumoral/edema area quantification of the different models represented as boxplots.

C) Magnetic Transfer Ratio (MTR)

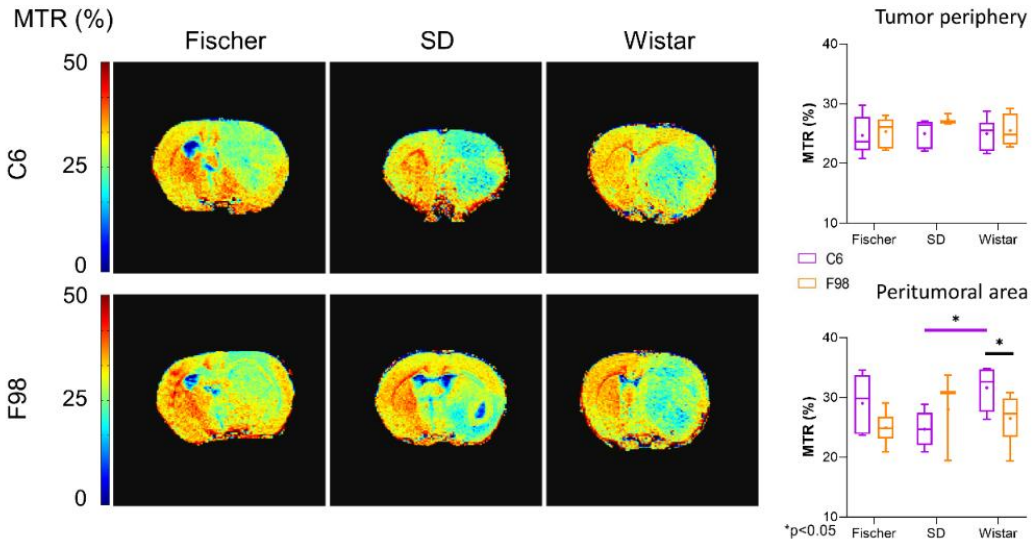

Figure S5. MTR maps analysis. Left: MTR maps from a representative rat of each ORM studied. Right: tumor periphery and peritumoral/edema area quantification of the different models represented as boxplots.

D) ^{1}H NMR Spectra

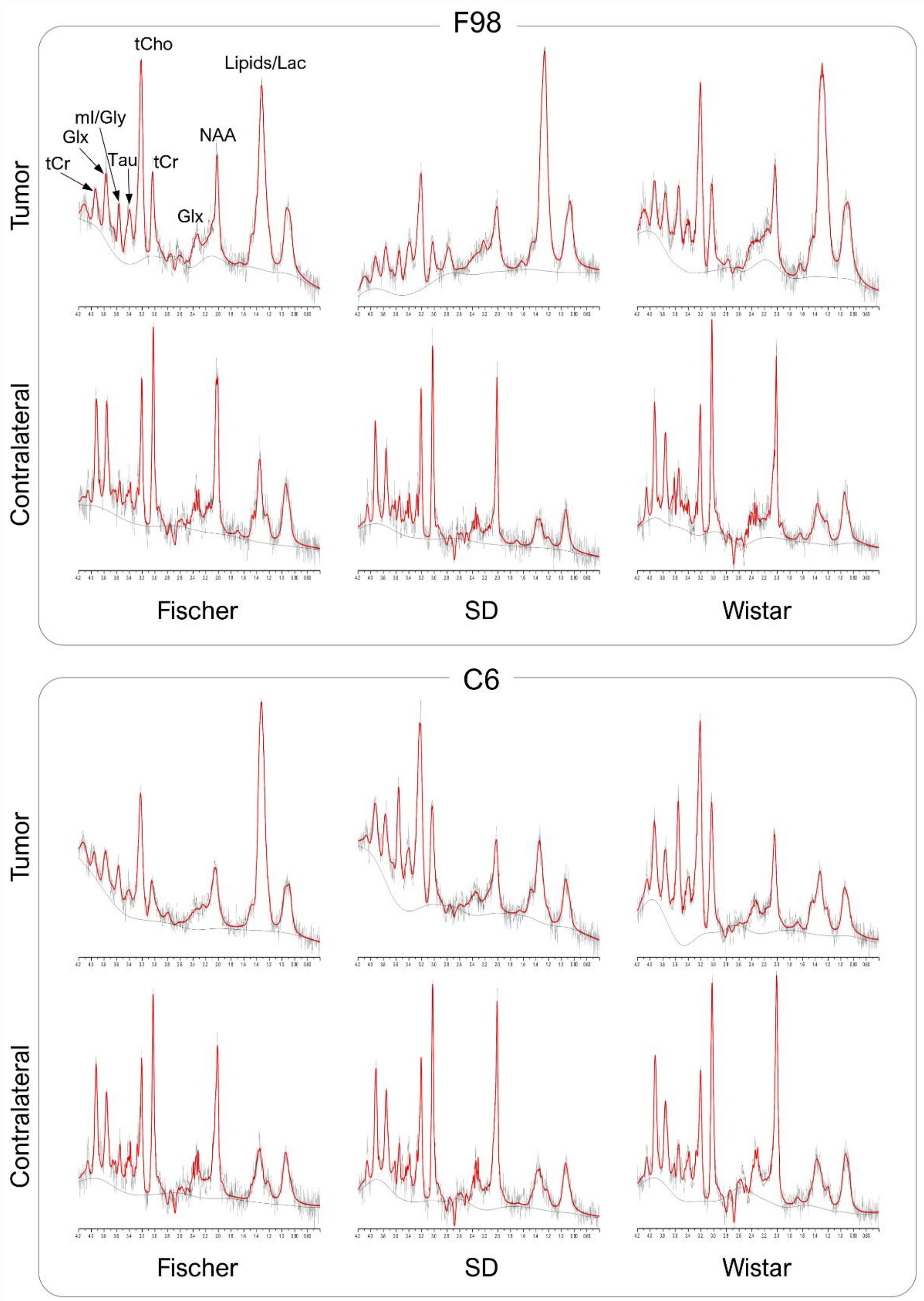

Figure S6. Representative *in vivo* ^{1}H NMR Spectra. Lipids/Lac: lipids+lactate, NAA: N-Acetylaspartate; Glx: glutamate+glutamine; tCr: total creatine (creatine+phosphocreatine); tCho: total choline (choline+phosphocholine+glycerophosphocholine); Tau: taurine; mI/Gly: myo-inositol+glycine.

References

1 Arias-Ramos, N. *et al.* Iron Oxide Incorporated Conjugated Polymer Nanoparticles for Simultaneous Use in Magnetic Resonance and Fluorescent Imaging of Brain Tumors. *Pharmaceutics* **13**, doi:10.3390/pharmaceutics13081258 (2021).

2 Guadilla, I., Gonzalez, S., Cerdan, S., Lizarbe, B. & Lopez-Larrubia, P. Magnetic resonance imaging to assess the brain response to fasting in glioblastoma-bearing rats as a model of cancer anorexia. *Cancer Imaging* **23**, 36, doi:10.1186/s40644-023-00553-y (2023).

3 Ortuno, J. E. *et al.* DCE@urLAB: a dynamic contrast-enhanced MRI pharmacokinetic analysis tool for preclinical data. *BMC Bioinformatics* **14**, 316, doi:10.1186/1471-2105-14-316 (2013).

4 Yankeelov, T. E. *et al.* Quantitative pharmacokinetic analysis of DCE-MRI data without an arterial input function: a reference region model. *Magn Reson Imaging* **23**, 519-529, doi:10.1016/j.mri.2005.02.013 (2005).

5 Provencher, S. W. Estimation of metabolite concentrations from localized in vivo proton NMR spectra. *Magnetic Resonance in Medicine* **30**, 672-679, doi:10.1002/mrm.1910300604 (1993).
